# Environmental and physiological correlates of the severity of clinical signs of snake fungal disease in a population of pigmy rattlesnakes, *Sistrurus miliarius*

**DOI:** 10.1093/conphys/cow077

**Published:** 2017-01-27

**Authors:** Ciera M. McCoy, Craig M. Lind, Terence M. Farrell

**Affiliations:** Department of Biology, Stetson University, Deland, FL 32723, USA

**Keywords:** Body condition, ecoimmunology, immune function, life history, *Ophidiomyces ophiodiicola*, trade-off

## Abstract

In the past decade, snake fungal disease (SFD) has been identified as an emerging threat to snake populations throughout the eastern USA. Snake fungal disease is caused by the fungus *Ophidiomyces ophiodiicola*. Little is known regarding the environmental or physiological variables that affect host vulnerability and *O. ophiodiicola* virulence in wild snake populations. Understanding the intrinsic and extrinsic factors that correlate with infection severity is a key first step in understanding host–pathogen dynamics. Host vulnerability may vary seasonally as a result of thermal conditions or energetic trade-offs, and pathogen growth rates or dispersal may be tied to seasonal trends in climate. To determine whether season, environmental temperature or energetic trade-offs associated with life-history stage influence an individual's susceptibility to infection, we monitored the severity of clinical signs of SFD, surface air temperature, reproductive status, body condition and serum complement activity (plasma bactericidal ability) in free-ranging pigmy rattlesnakes, *Sistrurus miliarius*, over the course of 18 months. Seasonal increases in the severity of clinical signs of SFD were correlated negatively with monthly air surface temperature and the mean body condition of the population. Bactericidal ability varied seasonally, but pigmy rattlesnakes suffering from active SFD infections did not exhibit deficits in innate immune function. Infected snakes were in significantly lower body condition when compared with the general population, but seasonal patterns in the mean body condition of the population were not driven by seasonal patterns of infection severity. Our results highlight the potential importance of the thermal environment and energetic status in determining infection severity and outcomes and the need for managers and researchers to consider seasonality of symptom presentation when the goal is to identify the prevalence or incidence of SFD in populations.

## Abstract (precis)

In free-ranging pigmy rattlesnakes, clinical signs of snake fungal disease varied seasonally and were negatively correlated with energetic status and mean air temperature. Severely infected snakes were in poor body condition but did not show deficits in innate immune function. Innate immunocompetence varied seasonally, but not in association with costly life-history stages.

## Introduction

Fungal pathogens have emerged as a major conservation concern in recent decades (Sutherland *et al*., 2014). Diseases such as white nose syndrome (WNS) in bats and chytridiomycosis in frogs have led to widespread population declines and a loss of global biodiversity (Blehert *et al*., 2009; Fisher *et al*., 2012). Recently, a skin disease (snake fungal disease; SFD) caused by the fungus *Ophidiomyces ophiodiicola* has become widespread in snake populations across North America (Allender *et al*., 2015; Lorch *et al*., 2016). Snake fungal disease was first tied to population decline in wild populations in 2006 and 2008 (Allender *et al*., 2011; Clark *et al*., 2011), and very little is known regarding the factors that influence individual and population-level outcomes of infection (Lorch *et al*., 2016). Predicting the future spread and impact of emerging pathogens is challenging and requires an understanding of intrinsic (physiological) and extrinsic (environmental) factors that affect host vulnerability and pathogen virulence (Altizer *et al*., 2006). Description of environmental and physiological correlates of infection severity in free-living populations is a first step towards identifying the factors that determine the spread and impacts of SFD and can inform effective management and conservation strategies (Berger *et al*., 2004; Rachowicz *et al*., 2005; Kriger and Hero, 2007; Kilpatrick *et al*., 2010).

Fungal infections often exhibit seasonality in wild populations because both host immune function and fungal growth rate are dependent on extrinsic factors, such as temperature (Nelson and Demas, 1996; Piotrowski *et al*., 2004; Kriger and Hero, 2007). Seasonal shifts in temperature may affect the physiological capability of individuals to fight infections (Gsell *et al*., 2013; James *et al*., 2015). In temperate endotherms, cold winter months are associated with both reduced food availability and increased costs of thermoregulation, which force trade-offs among processes key to self-maintenance (e.g. immune function and thermoregulation; Nelson and Demas, 1996). Ectotherms may face similar trade-offs if the amount of time and energy required to thermoregulate effectively fluctuates with season (Huey, 1974; Díaz and Cabezas-Diaz, 2004). If seasonal shifts in the thermal environment demand increased time–energy allocation to thermoregulation and food resources are limited, ectotherms may be forced to down-regulate immune function to maintain positive energy balance. Additionally, effective behavioural thermoregulation may be necessary for ectotherms to induce fever to defend against infections (Kluger *et al*., 1975). Therefore, seasonal fluctuation in ambient temperature may affect host defense by impairing the ability to induce fever, increasing thermoregulatory costs, or both.

Seasonal events in the life history of individuals may also lead to seasonal or sexual variation in infection rates and symptom severity (Saad and Elridi, 1984; Angilletta and Sears, 2000; Adamo *et al*., 2001; Schmid-Hempel, 2003; French *et al*., 2007a). Allocation toward immune function may be reduced in favour of allocation to energetically expensive life-history events, such as vitellogenesis and pregnancy in females or mate search and territory defense in males (Olsson *et al*., 2000; Verhulst *et al*., 2005; French *et al*., 2007a; Cox *et al*., 2010; Ruiz *et al*., 2010). If resources are allocated away from immune defense during seasonal reproductive events, hosts may be rendered vulnerable to opportunistic infections, thus driving seasonal patterns of infection (Olsson *et al*., 2000; Schmid-Hempel, 2003).

Seasonal shifts in prey availability may also affect time–energy trade-offs in populations. Seasonal scarcity of food resources or reduction in foraging success may lead to seasonal reductions in the size of individual energy budgets and may exacerbate trade-offs among competing life-history functions (e.g. growth and reproduction; Van Noordwijk and de Jong, 1986). If costly seasonal life-history stages coincide with increased thermoregulatory costs and/or seasonal declines in foraging success, individuals may allocate stored energy reserves away from immune defense to maintain positive energy balance, rendering hosts vulnerable to infection (Nelson and Demas, 1996; Boyles and Willis, 2010). Therefore, understanding infection risk and effective management of infected populations requires an understanding of the interplay among environmental variables (e.g. food availability and temperature), physiological variables (e.g. reproductive status and immunocompetence) and infection severity.

**Figure 1:**
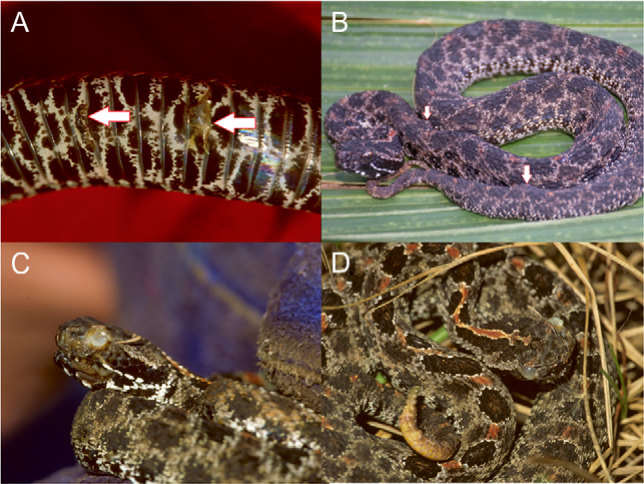
Example images of typical lesions on ventral surface (**A**), lesions on the dorsal surface (**B**) and a snake that was scored as a 3 (high) based on multiple body lesions in addition to lesions on the face (**C** and **D**). Lesions are indicated by arrows in A and B.

Examination of environmental and physiological correlates of infection in wild populations requires a model organism that is abundant, demonstrates high prevalence of infection and is accessible for sampling throughout the year. Pigmy rattlesnakes, *Sistrurus miliarius*, in Central Florida began to exhibit clinical signs consistent with SFD as early as 1998. A potential causative agent was cultured from lesions and identified as *Geotricum candidum* (Cheatwood *et al*., 2003), a fungus that is easily confused with *O. ophiodiicola* owing to similar morphology (Sigler *et al*., 2013). Recent molecular (real-time PCR) analyses have confirmed that *O. ophiodiicola* is present in the skin lesions observed in pigmy rattlesnakes in Central Florida (Lorch *et al*., 2016). Clinical signs of SFD are characterized by thickened, necrotic yellow-brown lesions on the skin of the head, body and tail (Fig. 1). Often, infected areas will show ulceration where lesions break off and swelling as the result of local immune responses (Lorch *et al*., 2015). Pigmy rattlesnakes are locally abundant and surface active throughout the year (May *et al*., 1996), which allows the annual relationships among intrinsic and extrinsic variables and symptom severity to be described more completely compared with hibernating species.

To gain a better understanding of the intrinsic and extrinsic variables that may affect host–pathogen dynamics and SFD infection outcomes, we scored the severity of clinical signs consistent with SFD infection (we cannot rule out the possibility that a causative agent other than *O. ophiodiicola* was resulting in clinical signs consistent with SFD) in a field-active population of pigmy rattlesnakes over the course of 18 months. We monitored air surface temperature, individual energetic status, reproductive status (females only) and the ability of plasma to kill a generic pathogen (a proxy for innate immune function) in individuals. We hypothesized that the severity of clinical signs of SFD would vary seasonally in association with seasonal shifts in the thermoregulatory and resource environment and in association with trade-offs between costly seasonal life-history stages and immune function. We therefore predicted that seasonal elevation in symptom severity in the population would be related to low environmental temperatures, costly reproductive life-history functions, energy limitation and impaired innate immune function.

## Materials and methods

### Field sampling

From 18 January 2015 to 20 June 2016, we scored the severity of fungal infection, sampled blood, determined reproductive condition and recorded mass in free-ranging adult pigmy rattlesnakes at Lake Woodruff National Wildlife Refuge (LW) in Volusia County, FL, USA. We made 537 observations on 257 individual snakes. We measured the snout–vent length of each snake using a squeeze box and weighed each individual in the field using a Pesola^®^ hanging spring scale. Each snake was marked by intraperitoneal injection of a PIT tag (Avid^®^, Norco, CA, USA). We drew a blood sample from the caudal vein within 5 min of initial contact using a 1 ml syringe and a 27-gauge needle. We placed each sample in a 1.5 ml microcentrifuge tube containing two drops of EDTA and placed it on ice for transport back to the laboratory within 4 h. Upon returning to the laboratory, we centrifuged the samples at 16 060 g for 10 min. We separated the red blood cells from plasma, and 30 μl of plasma were transferred to a 0.5 ml microcentrifuge tube for immunological testing. We stored samples at −80°C until they were used in assays.

### Variable calculation

We compiled monthly mean, minimal and maximal temperatures from data recorded at a local weather station and available online at MesoWest (29.10° latitude, 81.37° longitude; station number LWQF1). We scored the severity of SFD infection on a 0–3 point scale (Table 1 and Fig. 1). The scale was set at the start of the experiment, and all SFD scores were assigned by a single observer. Snakes that were assessed as between two levels were scored as half points in statistical analysis (i.e. a score of low/medium was recorded as 1.5). We calculated a body condition index (BCI) for each snake by taking the residual of a linear regression of logarithmically transformed mass on logarithmically transformed snout–vent length. We diagnosed the reproductive status of females by manual palpation of follicles and embryos in the field. Palpation was able to identify only large secondary follicles, and it is possible that we recorded some vitellogenic snakes as non-reproductive. We estimated innate immunocompetence via a bacterial killing assay using stored snake plasma.

**Table 1:** Classification of clinical sings of snake fungal disease infection (SFD score)

Clinical signs (SFD score)	Criteria
None (0)	No lesions present
Low (1)	Tail swelling and few lesions (<5) on ventral (Fig. 1A) or dorsal surface (Fig. 1B)
Medium (2)	Multiple lesions (>5) on dorsal and ventral surfaces, including tail (Fig. 1A and B)
High (3)	Lesions on face and/or cloaca as well as dorsal and ventral surfaces of the body (Fig. 1C and D)

### Assay procedures

We quantified the bacterial killing capacity of 190 plasma samples by using methods modified from Graham *et al*. (2011). We created a dilution using 3 μl of plasma and 97 μl of CO_2_ l-glutamine growth medium (CGM). We diluted *Escherichia coli* pellets (ACCT #8739; Microbiologics, St Cloud, MN, USA) in 40 ml of phosphate-buffered saline, and we incubated the solution for 1 h at 28°C. We further diluted the bacteria to a dilution of 1:64 and created test solutions using 20 μl of bacteria-containing solution with 100 μl of CGM (control) and 20 μl of bacteria-containing solution with 100 μl of the plasma/CGM solution (treated). After dilution and mixing, we allowed the solutions to react for 30 min at 28°C. We then spread 50 μl on duplicate agar plates using glass plating beads and incubated plates at 37°C overnight until colonies were clearly visible and able to be counted. We counted the number of colonies on each plate and calculated the proportion of bacteria remaining by dividing the mean number of colonies on treatment plates by the mean number of colonies on control plates. We then subtracted the proportion of colonies remaining from one to yield the proportion of bacteria killed (BKA score). We measured immune function in five separate assays. We used pooled plasma samples run in each of the assays to calculate an inter-assay coefficient of variation of 5%. We used duplicate samples to calculate an intra-assay coefficient of variation of 11.6%. When either plate was contaminated, we removed the entire sample from the analysis, leaving an assay-wide sample size of 175.

### Statistical analyses

All statistical analyses were conducted in JMP^®^, version 11 (SAS institute Inc., Cary, NC, USA). We examined seasonal variation in BCI within each sex by comparing the BCI scores of snakes sampled within each month by ANOVA. Tukey's *post hoc* tests were used to identify significant pairwise differences. We examined seasonal variation in the severity of clinical signs of SFD by ordinal logistic regression of month on SFD score. Pairwise differences were identified by comparing 95% confidence intervals of the parameter estimates for each month. We analysed relationships among average surface temperature, fungal score and BCI in each month by Pearson correlation. To examine innate immune function and to compare the physiological differences in snakes of differing fungal infection severity, we grouped the SFD score of individuals into three levels (none = 0, moderate = 0.5–1.5 and severe signs = 2–3). Given that the distribution of BKA scores violated the assumptions of parametric statistics, differences within each group were analysed by Kruskall–Wallace tests followed by Dunn's *post hoc* correction for multiple comparisons. The BCI of snakes at each level of infection was analysed by ANOVA followed by Tukey's *post hoc* tests. To analyse seasonal variation in innate immune function, we combined months into four meteorological seasons. Seasonal BKA scores were compared by Kruskall–Wallace test followed by Dunn's corrections for pairwise comparisons. We also compared the BKA scores in females during different stages of reproduction (non-reproductive, vitellogenic and pregnant) using non-parametric tests and compared SFD scores among different reproductive classifications by ordinal logistic regression. Our analysis includes some repeat sampling of individuals; however, repeat sampling was not extensive enough to use repeated-measures models. We therefore include repeated samples on individuals as independent observations. Individuals were marked with PIT tags and marked dorsally with nail polish to ensure that snakes were not sampled multiple times within the same 15 day period. Also, because encounters were random, only 15 out of 537 observations were taken from snakes that were sampled twice in the same month.

## Results

**Figure 2:**
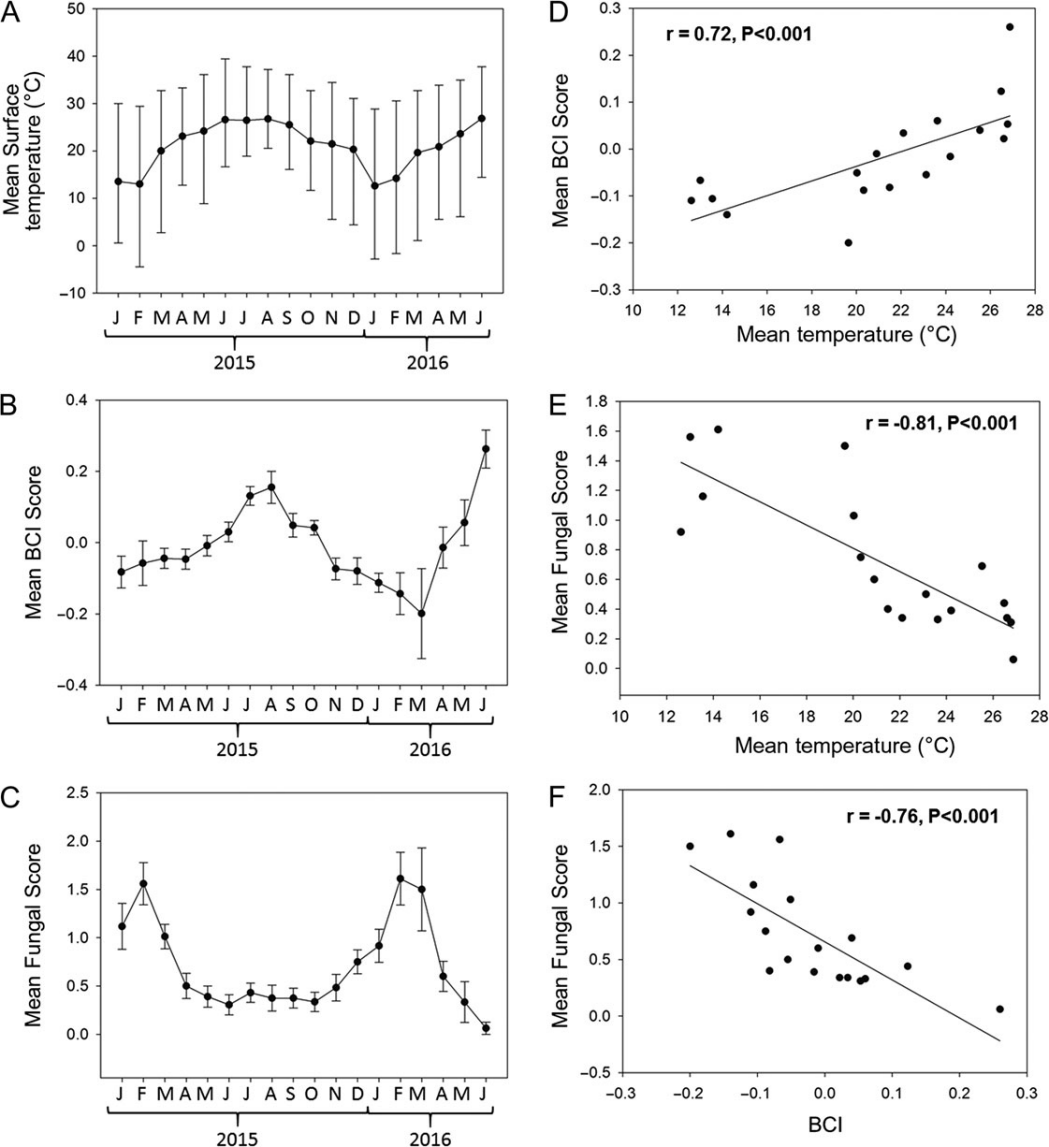
Monthly mean surface air temperature (**A**), body condition index (BCI; **B**) and snake fungal disease (SFD) score (**C**) for all 18 months of the study. Bars above and below means in A represent monthly minimal and maximal surface temperature. Bars in B and C represent the SEM. Linear correlations among the three variables are shown in **D–F**. Results of Pearson correlations are provided at the top of each graph.

**Figure 3:**
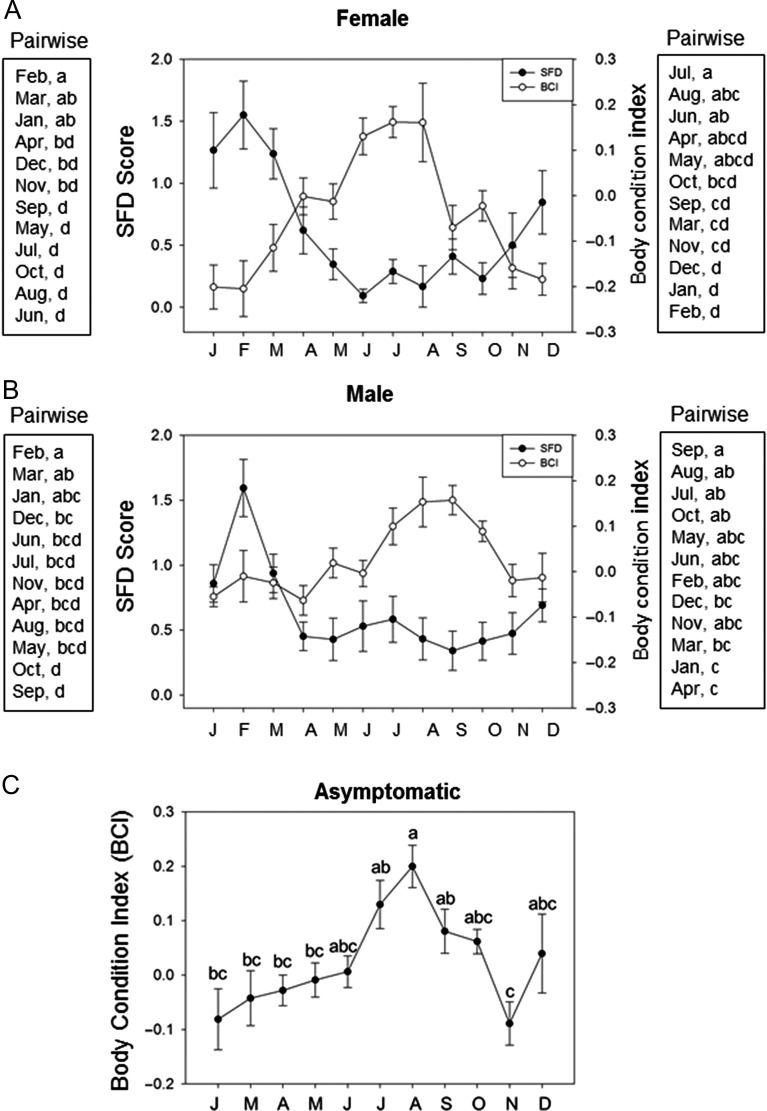
Annual trends in mean (±SEM) monthly body condition index (BCI) and snake fungal disease (SFD) score for females (**A**) and males (**B**) sampled in the study. The results of pairwise Tukey's HSD *post hoc* tests on monthly mean BCI are provided to the right of each axis. Pairwise differences in SFD score resulted from comparison of the 95% confidence intervals of the logistic regression parameter estimates for each month and are provided to the left of each axis. Months that do not share a letter are significantly different. Seasonal trends in the mean BCI of asymptomatic individuals in the population are shown in **C**. Pairwise Tukey's HSD results are indicated above the standard error bars for each month.

**Figure 4:**
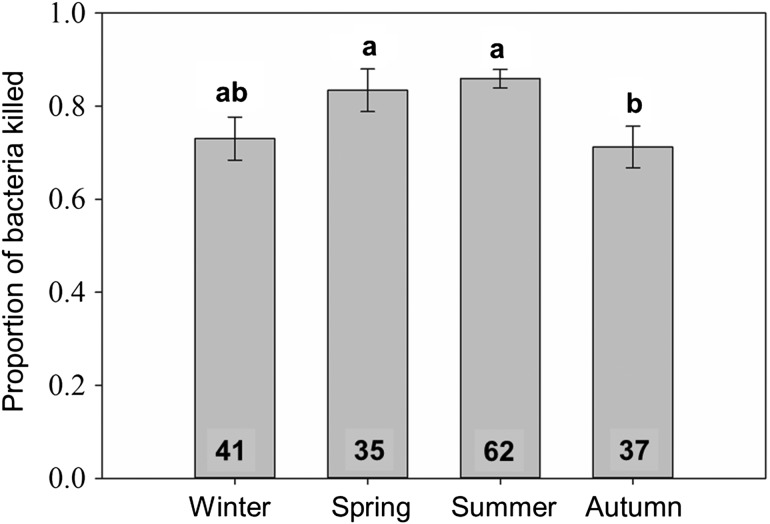
Mean±SEM BKA score (proportion of bacteria killed) in each season. Sample sizes are indicated at the base of each bar. Results of Dunn's *post hoc* tests are indicated above each bar. Seasons that do not share a letter are significantly different.

**Figure 5:**
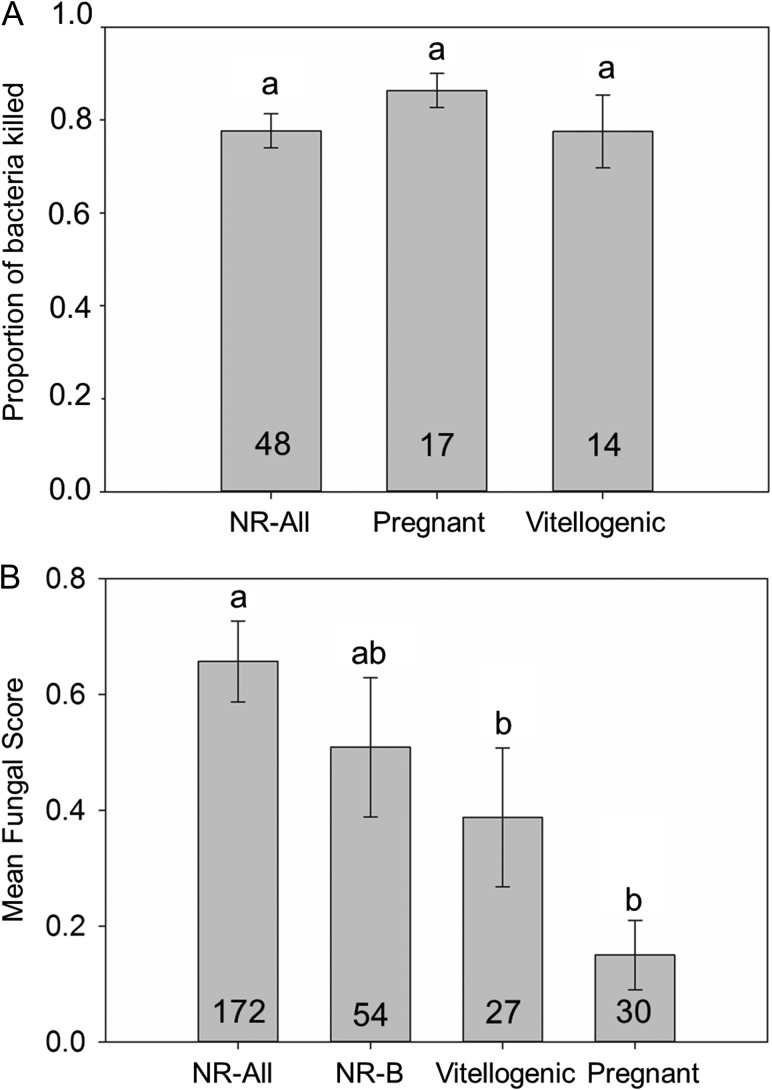
(**A**) BKA scores (proportion of bacteria killed) for each reproductive classification (NR-All = non-reproductive snakes sampled throughout the year). (**B**) Mean snake fungal disease (SFD) scores for reproductive (pregnant and vitellogenic) females compared with females sampled throughout the year (NR-All), and with only those non-reproductive females that were sampled during the breeding season (NR-B). *Post hoc* comparison of 95% confidence intervals on the logistic regression parameter estimates for each group are in indicated above each bar. All bars that do not share a letter were significantly different in *post hoc* comparisons. Sample sizes are indicated at the base of each bar.

**Figure 6:**
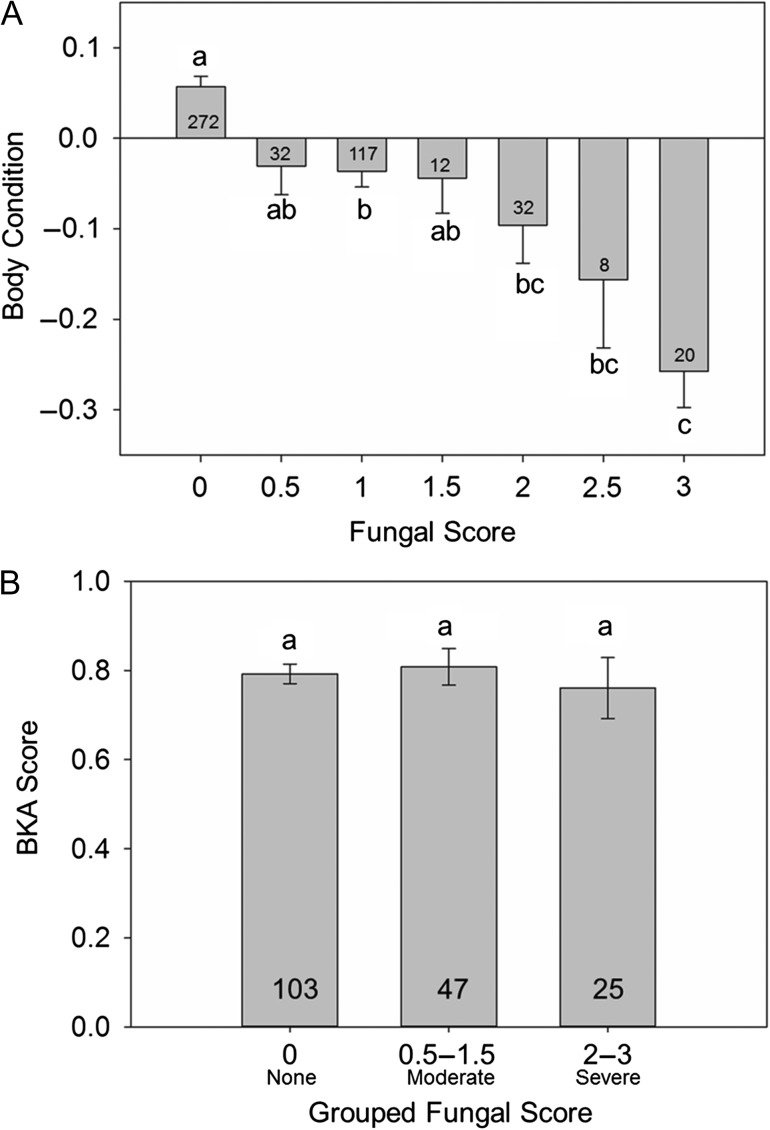
(**A**) Mean body condition index (BCI) of snakes scored at each of the seven possible snake fungal disease (SFD) scores. Results of Tukey's *post hoc* HSD tests are indicated by the letters above or below standard error bars. (**B**) Mean BKA score (proportion of bacteria killed) for each fungal classification (none, moderate or severe). Bars that share a letter are not significantly different. Sample sizes are indicated at the base of each bar.

The snout–vent length of sampled individuals ranged from 28.5 to 52.4 cm (mean ± SEM = 41.44 ± 0.18 cm). The mass of sampled individuals ranged from 21–147 g (mean = 62.81 ± 0.89 g). Monthly mean SFD scores were negatively correlated with both mean surface air temperature and mean BCI (Fig. 2E and F). Body condition index and mean surface air temperature were positively correlated (Fig. 2D). Female BCI and the severity of SFD both varied seasonally (ANOVA BCI, *F*_11,207_ = 7.76, *P* < 0.001; logistic regression SFD, χ^2^ = 65.36, *P* < 0.001; Fig. 3A). Male BCI and the severity of SFD also both varied seasonally (ANOVA BCI, *F*_11,262_ = 4.28, *P* < 0.001; logistic regression SFD, χ^2^ = 51.50, *P* < 0.001; Fig. 3B). The highest mean BCI was observed in months when the severity of infection was lowest (Figs 1 and 2). The seasonal pattern in BCI was similar when only asymptomatic snakes were analysed (Fig. 3C). Innate immunocompetence also varied seasonally (*H* = 14.54, *P* = 0.002), with snakes scoring lower on BKAs in the autumn compared with summer and spring (Fig. 4). Both vitellogenic and pregnant females displayed no significant deficits in innate immune function (*H* = 0.78, *P* = 0.68; Fig. 5A). Both pregnant and vitellogenic females displayed lower infection severity compared with all non-reproductive females (χ^2^ = 9.42, *P* = 0.01; Fig. 5B). However, differences were not significant when the analysis was restricted to non-reproductive females sampled during the breeding season (χ^2^ = 4.12, *P* = 0.13; Fig. 6B). Snakes with higher levels of infection had significantly lower BCI scores (*F*_2,458_ = 26.84, *P* < 0.001; Fig. 6A). Mean BKA scores were not significantly different among snakes with no, moderate or severe clinical signs of infection (*H* = 0.88, *P* = 0.65; Fig. 6B).

## Discussion

Our results support the hypothesis that the severity of clinical signs of SFD varies seasonally and in relationship to seasonal variation in air temperature and the mean energetic status (estimated by BCI) of the population (Fig. 2). Peaks in the severity of clinical signs of SFD in both males and females occur in months when mean energetic status and surface air temperature are lowest (Fig. 3). Additionally, individual snakes with moderate-to-severe fungal infection have a lower BCI compared with snakes with little or no evidence of infection (Fig. 6A). The relationship between energetic status and infection supports the hypothesis that energetic trade-offs affect host vulnerability. Our results do not support the hypotheses that costly life-history functions or compromised innate immune function are directly related to the increased symptom severity (Fig. 5). Innate immunocompetence varied seasonally, but not in direct relationship to the severity of fungal infection (Fig. 4).

Seasonal variation in infection severity suggests that seasonal shifts in biotic or abiotic factors alter either host vulnerability or pathogen virulence. The ability of ectothermic reptiles to fight infections often depends on the ability to up-regulate body temperature in response to infection (i.e. to induce fever behaviourally; Kluger *et al*., 1975; Casadevall, 2012; Cabañes *et al*., 2014). Previous studies conducted on pigmy rattlesnakes at our study site demonstrated that the cloacal temperatures of individuals track seasonal shifts in ambient temperature, suggesting seasonal shifts in either thermal preference or the ability to thermoregulate (May *et al*., 1996). The prevalence and severity of other emergent fungal pathogens, such as WNS and chytridiomycosis, also varies with seasonal thermal patterns (Sinclair and Lochmiller, 2000; Kriger and Hero, 2007; Boyles and Willis, 2010; Kilpatrick *et al*., 2010; Foley *et al*., 2011; Woodhams *et al*., 2011; Reeder *et al*., 2012; Langwig *et al*., 2015). Rates of WNS infection in bat populations show a seasonal pattern that is similar to SFD in our population. Rates of infection begin to rise in the late autumn and peak by late winter/early spring. Additionally, bats that survive winter routinely clear the infection by the summer months and even breed successfully (Langwig *et al*., 2015), just as we have observed in our population of pigmy rattlesnakes (our unpublished work). The pattern of infection in bats is attributed to both the spatial clustering of individuals and the physiological state of hibernating individuals (Langwig *et al*., 2015). Pigmy rattlesnakes do not hibernate in Central Florida and remain mostly solitary outside of the breeding season. Clark *et al*. (2011) found that an outbreak of SFD in a population of timber rattlesnakes, *Crotalus horridus*, was associated with extremely wet conditions. Such wet conditions could potentially exacerbate the spread of SFD infection by providing soil conditions conducive to fungal growth (Allender *et al*., 2015) or by impairing thermoregulation through increased cloud cover. In Central Florida, annual rainfall patterns follow a summer wet and winter dry seasonal pattern, thus the pattern of high signs of SFD in winter and low signs of SFD in summer observed in the present study suggests that increased rainfall and cloud cover do not drive increased severity of clinical signs of infection in winter months. We therefore find it likely that seasonal patterns in infection severity and the negative correlation between ambient temperature and infection severity observed in the population are the result of impaired thermoregulatory ability and/or increased costs (time and energy) of thermoregulation. An alternative explanation is that seasonal trends in infection severity may have nothing to do with the characteristics of the host, but are explained by the seasonal environmental variables that affect pathogen virulence or dispersal. The incidence of chytridiomycosis infection in frog populations, for example, has been shown to be correlated with monthly air temperatures, and seasonality in infection rates has been attributed to the thermal preferences of the fungus (Kriger and Hero, 2007). To our knowledge, no studies have investigated the thermal effects on the virlence of *O. ophiodiicola* (but see Allender *et al*., 2015). To determine the causal roots underlying seasonal trends in SFD infection, future experimental studies should examine thermal effects on both host and pathogen.

We predicted that female snakes would allocate resources away from innate immune function and exhibit increased infection severity during energetically expensive life-history events. Both pregnancy and vitellogenesis have associated costs in viviparous snakes (Van Dyke and Beaupre, 2011), and vertebrates often down-regulate aspects of immune function during costly reproductive events (reviewed by Martin *et al*., 2008). Such seasonal trade-offs have been demonstrated in reptiles, including snakes and lizards (French *et al*., 2007a,b; French and Moore, 2008; Graham *et al*., 2011). Our data do not support the hypothesis that such a trade-off occurs in pigmy rattlesnakes. Pregnant and vitellogenic snakes displayed no evidence of impaired innate immune function, and reproductive female snakes displayed lower average severity of fungal infection compared with non-reproductive females. Given that pigmy rattlesnakes often breed less than annually, and only snakes with ample stored energy enter reproductive bouts (Farrell *et al*., 1995), each year's cohort of reproductive females may represent the individuals in the population that have experienced the greatest foraging success and are in generally good health (i.e. are not suffering from fungal infection). Therefore, individuals that reproduce in a given year may be able to pay the costs of vitellogenesis and pregnancy without becoming more susceptible to infection. Reproductive effort may, however, leave females immunocompromised and vulnerable to opportunistic pathogens (Cox *et al*., 2010). A sharp drop in BCI was evident in the months after birth (September and October; Fig. 3A), and snakes sampled during the autumn scored significantly lower on bactericidal assays. Males may also experience increased concentrations of testosterone during the autumn because this time coincides with the onset of the breeding season, a life-history stage when elevated testosterone is observed in most crotaline snakes (Schuett *et al*., 1997, 2005; Taylor *et al*., 2004; Lind *et al*., 2010; Lind and Beaupre, 2015). Increased testosterone can be immunosuppressive and could render males more susceptible to infection at the onset of the breeding season (Roberts *et al*., 2004; Martin *et al*., 2008; Ruiz *et al*., 2010). The descriptive nature of our study precludes parsing causal effects of reproduction, reduced energetic status and falling ambient temperatures on immune function and infection severity in the autumn.

Time and energy trade-offs among costly functions can be exacerbated by shifts in individual demands for and access to energy (Van Noordwijk and de Jong, 1986). Seasonal patterns in the severity of SFD infection were negatively correlated with the mean BCI of the population (Figs 2F and 3), suggesting that seasonal shifts in resource availability may drive observed trends. A field study conducted at LW from 1993 to 2000 demonstrated that the foraging success of *S. miliarius* (estimated by the proportion of captures with a food bolus) was lowest in the months immediately preceding and coincident with lowest mean BCI in females and the highest infection severity observed in this study (from December to February; May and Farrell, 2012). Additionally, the BCI of infected snakes was negatively related to the severity of fungal infection, and infected snakes do not demonstrate evidence of impaired immune function (Fig. 6). In cases of WNS in bats, starvation during hibernation, rather than direct effects of the fungus itself, drives mortality. Hibernating bats increase energetic investment towards thermoregulation to fight the infection, enter negative energy balance and, ultimately, starve before food resources return in the spring (Boyles and Willis, 2010). In Central Florida, pigmy rattlesnakes do not hibernate. However, a seasonal decrease in the mean energetic status of uninfected snakes in the population is observed in the autumn and winter months when food resources are low and effective thermoregulation is likely to be impaired, perhaps causing reduced foraging success and/or increased costs during this time. It is possible that increased costs associated with winter thermoregulation, allocation to immune function to fight infection and decreased foraging success may drive mortality in pigmy rattlesnakes infected with SFD. Many severely infected snakes observed in the present study were extremely emaciated, and many were never recaptured (our unpublished data). Further research is required to establish the direct cause of mortality associated with SFD in wild snake populations.

Our descriptive study demonstrates several key points regarding the ecology of SFD in wild populations. First, presentation of clinical signs of SFD varies with season, with high prevalence and severity of infection in the cool months of winter and low infection severity in the summer. The high summer–low winter pattern was also observed in the mean BCI of males and females in the population. Severity of infection was not associated with costly reproductive life-history events or directly associated with impairment of innate immune function. Taken together, our results indicate that seasonal shifts in temperature and food availability may drive the observed seasonal trends. Our findings also point out several gaps in our understanding of the ecology of SFD. For example, we cannot eliminate the possibility that trends are driven by the ecology and physiology of the fungus itself. To model the outcomes of SFD infection effectively in wild snake populations, experimental work must be done to establish the causal variables that underlie both host vulnerability and pathogen virulence.

Conservation biologists and managers must take the seasonality of SFD infection severity into account when their goal is to document infection rates in the population, as sampling at different times of the year will yield drastically different estimates. For example, 100% of snakes sampled in February (2015 and 2016) presented clinical signs of SFD in this study compared with 25% in August (2016). To rule out the potential for other fungal pathogens to affect the presentation of clinical signs of SFD, the extreme seasonality in the estimated severity of clinical signs must also be verified by molecular (PCR) analysis of skin flora to establish the seasonal prevalence of *O. ophiodiicola*. Although cause-and-effect relationships between thermal environments or individual energetic status and host vulnerability have yet to be established, manipulation of thermoregulatory environments and food supplementation may prove to be successful rehabilitation strategies for infected individuals, particularly in at-risk species.
